# Sevoflurane reduces lipopolysaccharide-induced apoptosis and pulmonary fibrosis in the RAW264.7 cells and mice models to ameliorate acute lung injury by eliminating oxidative damages

**DOI:** 10.1080/13510002.2022.2096339

**Published:** 2022-07-08

**Authors:** Fushuang Zheng, Xiuying Wu, Jin Zhang, Zhiling Fu, Yan Zhang

**Affiliations:** a Department of Thoracic Surgery, Shengjing Hospital of China Medical University, Shenyang, People’s Republic of China; b Department of Anesthesiology, Shengjing Hospital of China Medical University, Shenyang, People’s Republic of China

**Keywords:** Sevoflurane, lipopolysaccharide, oxidative stress, cell apoptosis, pulmonary fibrosis

## Abstract

**Objectives:**

Sevoflurane is identified as an effective candidate drug for acute lung injury (ALI) treatment, but its curing effects and detailed mechanisms have not been fully disclosed. The present study was designed to resolve this academic issue.

**Methods:**

The ALI mice models were established, and Hematoxylin-eosin staining assay was performed to examine tissue morphologies. Cell viability was determined by CCK-8 assay, and Annexin V-FITC/PI double staining assay was used to examine cell apoptosis. The expression levels of proteins were determined by performing Western Blot analysis and immunofluorescence staining assay. ROS levels were examined by using DCFH-DA staining assay.

**Results:**

In this study, we investigated this issue and the ALI models were respectively established by treating the BALB/c mice and the murine macrophage cell line RAW264.7 with different concentrations of lipopolysaccharide (LPS) in vivo and in vitro, which were subsequently subjected to sevoflurane co-treatment. The results showed that sevoflurane reduced LPS-induced ALI in mice and suppressed LPS-triggered oxidative stress and apoptotic cell death in the RAW264.7 cells. Interestingly, we evidenced that the elimination of reactive oxygen species (ROS) by N-acetyl-L-cysteine (NAC) reversed LPS-induced cell apoptosis in RAW264.7 cells. Then, we verified that sevoflurane suppressed oxidative damages to restrain LPS-induced apoptotic cell death in the RAW264.7 cells through activating the anti-oxidant Keap1/Nrf2 pathway. Mechanistically, sevoflurane down-regulated Keap1 and upregulated Nrf2 in nucleus to activate the downstream anti-oxidant signaling cascades, which further ameliorated LPS-induced cell apoptosis and lung injury by eliminating oxidative damages.

**Discussion:**

Taken together, our study illustrated that the sevoflurane attenuates LPS-induced ALI by inhibiting oxidative stress-mediated apoptotic cell death and inflammation, and the Keap1/Nrf2 pathway played an important role in this process.

## Introduction

Acute lung injury (ALI) and its resultant acute respiratory distress syndrome are defined as severe clinical illness, and the mortality of this disease reaches about 26–58%, which is a huge concern for public health each year [1]. Recent data suggested that oxidative stress and excessive inflammation are two important contributors for ALI progression [2]. ALI may cause a series of direct or indirect impacts, including pneumonia and other inflammation-associated diseases, such as acute cardiac insufficiency, renal insufficiency, liver dysfunction, and life-threatening [3–5]. Internally, ALI will cause redox imbalance, oxidative damages, and DNA damage resulting in the death of the lung cells and dysregulated pulmonary functions [6]. Lipopolysaccharide (LPS) is one of the most important pathogenetic antigens of gram-negative bacteria, which is usually used to establish the bacteria infection-caused ALI in the previous studies [7]. As previously described, LPS triggers super-inflammation, promotes leukocyte infiltration, and seriously damages the normal functions of the the lung [8], which further facilitate second damage to the alveoli and aggravate the development of ALI [9,10]. According to the above information, it becomes urgent to search for the ideal agents which are effective to ameliorate LPS-induced inflammation and oxidative damages-associated cell death in ALI.

The volatile anesthetic sevoflurane is used as the first-line anesthesia, which is featured with low toxicity and pungency [11]. In addition to its narcotic properties, sevoflurane has been demonstrated to exert organs protection in patients [12], and researchers report that inhaled sevoflurane suppresses cell death to exert its protective effects in cerebral, renal, cardiac, and lung-associated diseases [13–15]. Several reports indicated that sevoflurane could attenuate lung injury. It is widely believed that sevoflurane attenuates lung injury by inhibiting inflammation response after reperfusion injuries. And a lot of experiments investigate that sevoflurane improves LPS-induced ALI by inhibiting lung inflammatory responses [16–18]. And some studies also show that sevoflurane alleviates organ reperfusion injury by decreasing oxidative stress. For example, Wanger et al. [19] show that sevoflurane posttreatment prevented oxidative in ventilator-induced lung injury, and Luo et al. [20] indicate that sevoflurane exerts anti-oxidant effects to ameliorate hypoxia-induced lung injury. However, the mechanism of the effect of sevoflurane on reducing oxidative response and then attenuating ALI is unclear.

The factor erythroid 2-related factor 2 (Nrf2) is identified as a classical anti-oxidant agent in various types of diseases [21]. Mechanistically, Nrf2 binds to Kelch-like ECH-related protein 1 (Keap1) to form the Keap1-Nrf2 complex in the cytoplasm in normal circumstances, and the Nrf2 is relived from the complex to translocate from cytoplasm to nucleus, which further interacts with the antioxidant response elements to achieve its anti-oxidant effects by promoting the transcription of heme Oxygenase-1 (HO-1) and NAD(P) H: quinone oxidoreductase 1 (NQO1) [22]. Among those genes, HO-1 inhibits inflammatory responses in cells by catalyzing the breakdown of heme [23]. During this process, heme is converted into three bioactive products namely free iron, carbon monoxide, and biliverdin, which is rapidly converted to bilirubin and plays crucial roles in inflammation, apoptosis, and oxidative stress [24]. And NQO1 is promoted by Nrf2, which functions to control inflammation and limit oxidative stress [20]. It has been reported that Nrf2 mediates redox regulation through multiple mechanisms. Since the mitochondrion is a primary site for ROS production under physiological and pathological conditions, a series of studies demonstrated the crucial role of the Nrf2 signaling pathway in cytosolic and mitochondrial ROS production. A recent report shows that Nrf2 is associated with the outer mitochondrial membrane and protects mitochondria from oxidative stress, whereas this protective effect was absent in Nrf2 knockout mice [25]. Nrf2 directly modulates the ROS and RNS by regulating the levels of superoxide and peroxides through the induction of antioxidant enzymes such as superoxide dismutase (SOD), glutathione peroxidase (GSH-Px), and peroxiredoxin (Prx) members [26]. In addition to this, Nrf2 also plays a role in the regulation of autophagy, iron storage, and metallothionein. Hence, in response to oxidative stress, up-regulation of Nrf2 signaling activates a complex antioxidant response and maintains the redox homeostasis through the regulation of multiple mechanisms.

Many studies have suggested that regulation of Keap1-Nrf2 signaling pathways can affect the development of lung injury. Specifically, Huang et al. [27] suggest that this pathway regulates endoplasmic reticulum stress, apoptosis, and autophagy to attenuate the LPS-induced ALI. Hu et al. [28] demonstrate that the Nrf2-Keap1-antioxidant response element (ARE) signaling pathway can eliminate oxidative stress and its associated inflammation to improve traumatic lung injury-related pathology, and a Keap1-Nrf2 inhibitor designed by Zhang et al. is expected to be a novel protective agent of ALI [29]. In addition, it has been reported that some drugs and compounds can alleviate lung injury by regulating Keap1-Nrf2 pathways, such as ginsenoside Rh2 [30], sinomenine [31], and panaxydol [32]. However, whether sevoflurane attenuates oxidative stress and subsequent inflammation responses via the Keap1-Nrf2 pathway has not yet been investigated.

Collectively, this study aimed to investigate the role and mechanisms of sevoflurane in protecting against oxidative stress-induced ALI by animal and cell model. This study from *in vivo* and *in vitro* experiments found that sevoflurane could attenuate ALI by inhibiting ROS generation as the same as N-acetylcysteine (NAC). And Keap1-Nrf2 pathway may involve in the anti-oxidative stress process of sevoflurane.

## Materials and methods

### Establishment of mice ALI model and treatment

The male BALB/c mice (8–12 weeks old, 20–30 g) were obtained from the Research Animal Center of China Medical University and were fed in the standard conditions with the temperature at 22 ± 2°C, the light–dark circle with 12 h interval and 40–60 humidified air. To establish the ALI model of mice, the mice were intratracheally treated with 1, 2.5, 5 or10 mg/kg lipopolysaccharide (LPS, Escherichia coli serotype 055: B5; Sigma-Aldrich, Milwaukee, Wisconsin; Merck KGaA, Darmstadt, Germany). The mice which were treated with 0.3 ml saline treatment were used as negative control (NC). Then, the mice were subjected to 3% sevoflurane (United States Pharmacopeia Reference Standard, Sigma-Aldrich; Merck KGaA) for 4 h, starting 2 h after LPS treatment. After the above-mentioned administration, the animals were euthanized by CO_2_ asphyxiation [33]. All the animal experiments were approved by the Ethics Committee of Shengjing Hospital of China Medical University in August 2020 and the approval number was No. 2020-KY-0523.

### Hematoxylin–eosin staining

The right lung of the mice was fixed with 4% paraformaldehyde, prepared as slices with 4 μm thickness, and a commercial hematoxylin–eosin staining kit (Solairbio, Beijing, China) was used for histopathological analysis, the detailed experimental procedures have been documented in the manufacturer’s protocol. The degree of lung damage was assessed by using a light photomicroscope, which revealed alveolar edema, hemorrhage, alveolar septal thickness, and polymorphonuclear leukocytes infiltration.

### Lung wet/dry weight ratio

The wet (W) and dry weights of the left lung were determined before and after 48 h of drying at 65°C. Then calculating the wet/dry weight ratio yielded the water content.

### Evaluation of lung permeability

The Evans blue dye extravasation method was used to determine lung permeability, as previously referenced. Before the mice were euthanized, 5% Evans Blue stain (Solaibio) was injected through the tail vein for 10 h. The lung was then fully perfused with saline buffer solution and was treated with formamide to extract the Evans blue. The supernatants were collected and analyzed by using a microplate reader (Thermo Fisher Scientific Inc., Waltham, MA) at the absorbance of 620 nm. The lung permeability was calculated by inferring to the Evans blue per tissue weight (µg/g).

### Culture and treatment of the cells

The murine macrophage cell line RAW264.7 (ATCC, Manassas, VA, USA) was grown in Roswell Park Memorial Institute (RPMI)-1640 medium (Procell, Hubei, China) containing 10% fetal bovine serum (FBS). The RAW264.7 cells were then treated with 0.1, 0.5, 1, or 5 μg/ml LPS for 2 h at 37°C to establish cellular ALI models. To observe the effect of oxidative stress on LPS-induced ALI, oxidative inhibitor acetylcysteine (NAC, Sigma-Aldrich; Merck KGaA) was used at a concentration of 1, 2, 5 or 10 nM which dissolved in sterile deionized water for 30 min before LPS treatment [34]. And to observe the antioxidant effect of sevoflurane on ALI, 2 h after LPS stimulation, RAW264.7 cells were exposed to 3% of sevoflurane for 4 h at 37°C as referred to the method by Steurer et al. [35].

### RNA interference of Nrf2 and overexpression of Keap1

ShRNA specific for mouse Nrf2 (shRNA-GFP-Nrf2) and overexpression of Keap1 (pcDNA3-GFP-Keap1) plasmids were purchased from Sangon Biotech (Shanghai, China). The expression plasmids were transfected into RAW264.7 cells using the X-tremeGENE HP DNA transfection reagent (Roche Applied Science, Basle, Switzerland). After culturing for 24 h with a medium containing the transfection reagent, the cells were used to confirm the overexpression of Keap1 or Nrf2 by Western Blot. For in vivo transfection, an HVJ envelope-vector kit (GenomONE, Ishihara-Sangyo Kaisha Ltd., Osaka, Japan) was used, according to the manufacturer's instructions [36]. Administration of the HVJ envelope vector containing the shRNA-GFP-Nrf2 plasmid or pcDNA3-GFP-Keap1 (8 μg) was performed at the same time as LPS. Nrf2 inhibition or Keap1 overexpression vector solution was injected under anesthesia at the location of the lung. An empty vector was injected into the corresponding area as a control in the same way.

### Examination of cell viability

The Cell Counting Kit-8 (CCK-8, Beyotime, Shanghai, China) was purchased to evaluate cell viability, and the RAW264.7 cells were cultured into a 96-well microplate (5 × 10^3^ cells/well) and treated with LPS, sevoflurane, and/or transfected. A total of 10 μl of CCK-8 reagent was applied to the microplates for 2 h incubation at 37°C. A microplate reader (Thermo Fisher Scientific Inc.) was employed to examine the absorbance at 450 nm, which represented the relative cell viability.

### Cell apoptosis assay

An Annexin V-FITC cell apoptosis detection kit (Beyotime, Shanghai, China) was used to determine cell apoptosis in accordance with the manufacturer’s procedure. The RAW264.7 cells were treated with LPS, sevoflurane and/or transfected, and 1 × 10^5^ cells were then collected and diluted into Annexin V-FITC binding buffer. Following that, cells were incubated with Annexin V-FITC and propidium iodide (PI) shielded from light for 15 min at room temperature. A flow cytometer (BD Biosciences, San Jose, CA, USA) and FlowJo software version 7.6 (FlowJo, TreeStar, Ashland, OR, USA) were used for the analysis of the flow cytometric.

### Evaluation of oxidative stress status

Referring to the methods of Sun et al. [37], the lung tissues were homogenized with RIPA lysis buffer on ice, and the homogenate was subjected to centrifugation at 2000 rpm for 10 min, and the supernatants were collected and stored. Also, the RAW264.7 cells were treated with LPS and/or sevoflurane and processed as suspension. The ROS, MDA, SOD, glutathione (GSH), and oxidized glutathione (GSSG) were quantified using commercial assay kits (Jiancheng Bioengineering Institute, Nanjing, China) according to the manufacturers’ procedure. A microplate reader (Thermo Fisher Scientific Inc.) was used to evaluate the activity of their activities. To visualize the ROS level in cells, DCFH-DA staining was observed and showed using a fluorescence microscopy.

### Isolation of nuclear and cytoplasmic extract

RAW264.7 cells with transfection or without transfection were lysed using RIPA lysis buffer. After the cells were vortexed gently for lysis, the mixture was subjected to 3000 rpm centrifugation for 15 min to remove the cell debris. Nuclear-cytoplasmic fractionation was conducted using the NE-PERTM Nuclear and Cytoplasmic Extraction Reagents (Thermo Fisher Scientific Inc.) in keeping with the producer’s protocol.

### Western Blot

Protein extraction was conducted by using the RIPA lysis buffer (Solarbio) and the BCA method was employed to ensure protein purity. 10% SDS-PAGE was used to separate the proteins according to their molecular weight, and the target proteins were transferred onto the PVDF membranes (Millipore, Boston, MA). The membranes were then blocked, and probed with primary antibodies, including cleaved Caspase-3 (#ab2302), B-cell lymphoma-2 (Bcl-2, #ab182858), Bcl-2 Associated X (Bax, #ab32503), Keap1, (#ab119403), Nrf2, (#ab62352), HO-1, (#ab68477), SOD1, (#ab13498), NQO1, (#ab80588), β-actin (#ab115777), or Lamin A (#ab133256) in blocking buffer at 4°C overnight. Then, the membranes were incubated with horseradish peroxidase-conjugated goat anti-rabbit IgG (1:10,000; Abcam, #ab205718) for 60 min in the dark, and developed by enhanced chemiluminescent reagent (#32209, Thermo Fisher Scientific, Inc.). The membrane was exposed to a chemiluminescence apparatus (Bio-Rad Laboratories, Inc., Hercules, CA, USA), and the visualized protein bands were analyzed by the Image J software (NIH, Washington D.C.).

### Immunofluorescence staining

The nuclear translocation of Nrf2 was investigated using immunofluorescence staining. In brief, the formalin-fixed RAW267.4 cells were incubated with the anti-Nrf2 antibodies at 4°C overnight, which were further washed with PBS buffer to remove the residual antibodies. Then, the cells were blocked by the 1% blocking solution and incubated probed with the fluorescein-conjugated secondary antibody. Following that, the nucleus was stained with DAPI at the concentration of 1 μg/ml for 10 min, and the fluorescence signals were observed and analyzed by using a fluorescence microscope (ZEISS, Oberkochen, Germany) at the magnification of 200×.

### Statistical analysis

All the data was collected and presented as Means ± SD, and we employed the SPSS 19.0 software (IBM, Armonk, NY, USA) for statistical analysis. Specifically, the means in two groups were compared by performing the student’s t-test and a one-way ANOVA analysis was performed to analyze the statistical significance of the data from multiple groups above 3. *P* < 0.05 was considered as significantly different and were marked by ‘*’.

## Results

### LPS induces pulmonary fibrosis and apoptotic cell death in ALI mice models

As previously reported, we initially established the animal models for ALI by administering the male BALB/c mice with differential concentrations of LPS (0, 1, 2.5, 5, and 10 mg/kg), and the mice lung tissues were obtained. The H&E staining assay results in Figure S1A and S1B showed that LPS significantly altered mice pulmonary tissue morphology from normal status to fibrosis, and promoted mice lung injury. Also, we showed that LPS promoted inflammatory cells infiltration and thickened the alveolar walls in mice lung tissues (Figure S1A). Subsequently, we verified that the lung wet/dry ratio and Evans blue permeability was significantly increased by LPS treatment in a concentration-dependent manner (Figure S1C and S1D). In addition, given that cell apoptosis is determined as a pivotal contributor for LPS-induced ALI, we investigated this issue by determining the expression status of the key genes that regulated cell apoptosis in mice lung tissues, and the results showed that LPS concentration dependently increased the expression levels of pro-apoptotic proteins (cleaved Caspase-3 and Bax), whereas suppressed the anti-apoptotic Bcl-2 protein in mice lung tissues (Figure S1E). Those data suggested that LPS caused lung fibrosis and promoted cell apoptosis in the ALI mice models *in vivo*.

### The promoting effects of LPS treatment on oxidative damages and apoptosis in the RAW264.7 cells *in vitro*

**Figure 1.**
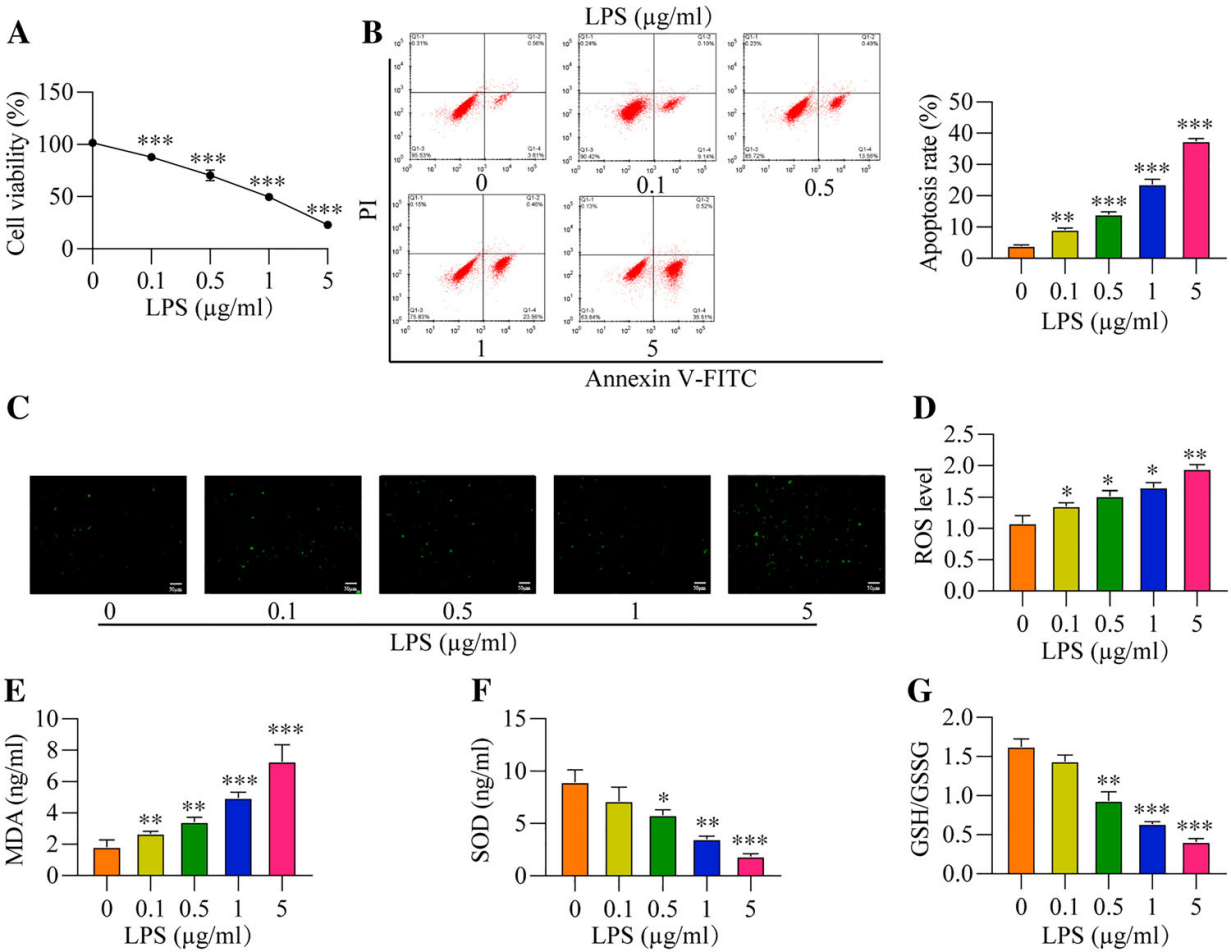
LPS induces cytotoxicity and oxidative stress *in vitro*. After different concentration of LPS treatment (0, 0.1, 0.5, 1, and 5 μg/ml) for 2 h, all cells were collected and prepared for suspension. And then, (A) Cell viability, (B) Apoptosis rate, (C) DCFH-DA staining, (D) ROS level, (E) MDA level, (F) SOD level, and (G) GSH/GSSG ratio were measured, respectively. All data were presented as the mean ± SD. N = 4 in each group. *P < 0.05, **P < 0.01 and ***P < 0.001 vs. 0 μg/ml LPS group. LPS, Lipopolysaccharide. ROS, reactive oxygen species. MDA, malondialdehyde. SOD, superoxide dismutase. GSH, glutathione. GSSG, oxidized glutathione.

To observe the effects of LPS on cell cytotoxicity and oxidative stress, the RAW264.7 cells were administered with increasing concentrations of LPS (0, 0.1, 0.5, 1 and 5 μg/ml). As shown in Figure 1(A), LPS dose-dependently decreased cell viability in the RAW264.7 cells, and the IC_50_ for LPS was 0.8487 μg/ml. Consistently, we performed the flow cytometry analysis to evaluate cell apoptosis and expectedly found that LPS also increased the cell apoptosis ratio in the RAW264.7 cells (Figure 1(B)). Then, we verified that LPS also triggered oxidative damages in the RAW264.7 cells (Figure 1(C–G)). Specifically, as shown in Figure 1(C and D), the DCFH-DA staining and corresponding ROS level were increased with LPS concentration. Also, the MDA levels were increased with LPS concentration (Figure 1(E)), while SOD levels were decreased (Figure 1(F)). The GSH/GSSG ratio was an important indicator of oxidative stress [38], and we evidenced that the GSH/GSSG ratios were reduced by LPS treatment (Figure 1(G)). In addition, the mitochondria ROS level also decreased with LPS concentration (Figure 1(H)). The above results indicated that LPS caused oxidative damages and cell apoptosis in the RAW264.7 cells *in vitro*. Moreover, according to the results in Figure 1 and Figure S1, we selected the 1μg/ml of LPS to treat RAW264.7 cells for the following experiments.

### LPS treatment contributes to cell apoptosis in the RAW264.7 cells via triggering excessive oxidative damages

According to the previous publications, there exist interplays between oxidative stress and cell apoptosis, and excessive oxidative damages have been reported to cause apoptotic cell death. Since we have evidenced that LPS-induced both oxidative stress and cell apoptosis in the RAW264.7 cells, we next explored their detailed relationship. To achieve this, the RAW264.7 cells were respectively treated with 1μg/ml of LPS and increasing concentration of ROS scavenger NAC (0, 1, 2, 5, 10 nm). Interestingly, our data showed that LPS significantly suppressed cell viability in the RAW264.7 cells, which were does-dependently increased by NAC co-treatment (Figure S2A). Consistently, the apoptosis ratio of the cells was determined, and we verified that the promoting effects of LPS treatment on the RAW264.7 cell apoptosis were also reversed by NAC in a concentration-dependent manner (Figure S2B). The above data hinted that LPS promoted apoptotic cell death in the RAW264.7 cells by triggering excessive oxidative damages.

### Sevoflurane alleviates LPS-induced lung fibrosis and oxidative stresses *in vivo*

**Figure 2.**
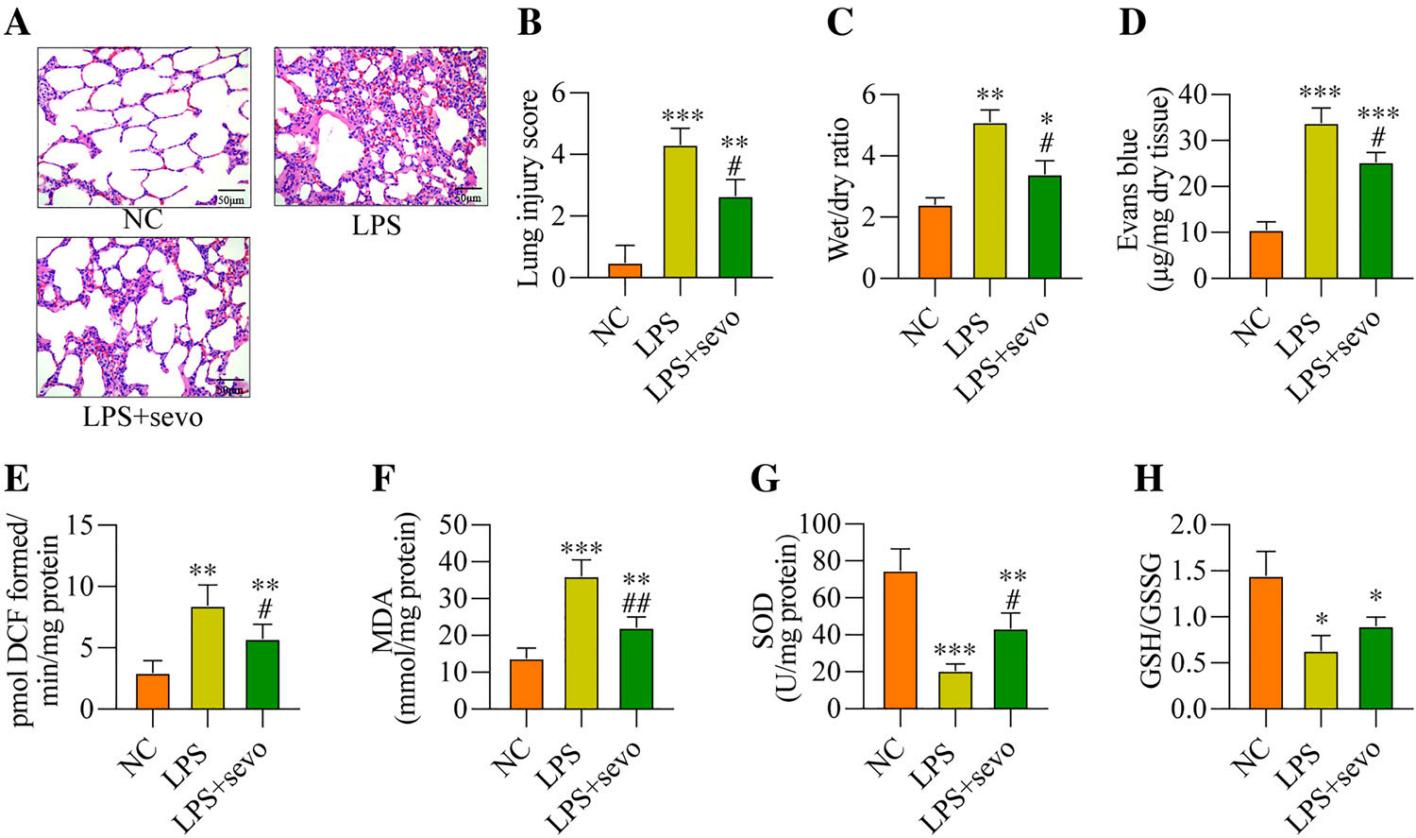
Sevoflurane alleviates LPS-induced ALI and oxidative stress *in vivo*. All mice treated with 5 mg/kg LPS for 2 h and/or administered 3% sevoflurane for 4 h, the lung tissues were collected. (A) The histology of lung tissues, (B) lung injury score, (C) wet/dry of lung tissues, (D) permeability of lung vasculature, (E) ROS generation, (F) MDA level, (G) SOD level, and (H) GSH/GSSG ratio were measured, respectively. All data were presented as the mean ± SD. N = 6 in each group. **P < 0.01 and ***P < 0.001 vs. NC group. #*P* < 0.05 and ##*P* < 0.01 vs. LPS group. NC, negative control. LPS, Lipopolysaccharide. Sevo, sevoflurane. ROS, reactive oxygen species. MDA, malondialdehyde. SOD, superoxide dismutase. GSH, glutathione. GSSG, oxidized glutathione.

Next, to ask whether sevoflurane-regulated oxidative damages in LPS-induced ALI models, the mice were treated with 5 mg/kg LPS and were administered with 3% sevoflurane, and the mice pulmonary tissues were collected for H&E staining assay, which showed that LPS-induced lung fibrosis and injury in ALI mice were partially ameliorated by sevoflurane co-treatment (Figure 2(A, B)). Also, sevoflurane decreased the lung wet/dry ratio (Figure 2(C)) and the permeability of lung vasculature (Figure 2(D)) in the LPS-treated mice lung tissues in contrast with the LPS alone group, indicating that sevoflurane treatment could ameliorate LPS-induced ALI in mice. Then, the antioxidant effects of sevoflurane in LPS-induced ALI mice models were investigated, and we expectedly found that sevoflurane reduced ROS (Figure 2(E)) and MDA levels (Figure 2(F)), while enhanced the SOD levels (Figure 2(G)) in the LPS-treated mice lung tissues. Consistently, the GSH/GSSG ratio was also increased with sevoflurane treatment in the LPS-induced ALI mice tissues (Figure 2(H)). The above results demonstrated that sevoflurane treatment could reduce oxidative stress in ALI mice.

### Sevoflurane suppresses LPS-induced cell apoptosis and oxidative damages in the RAW264.7 cells *in vitro*

**Figure 3.**
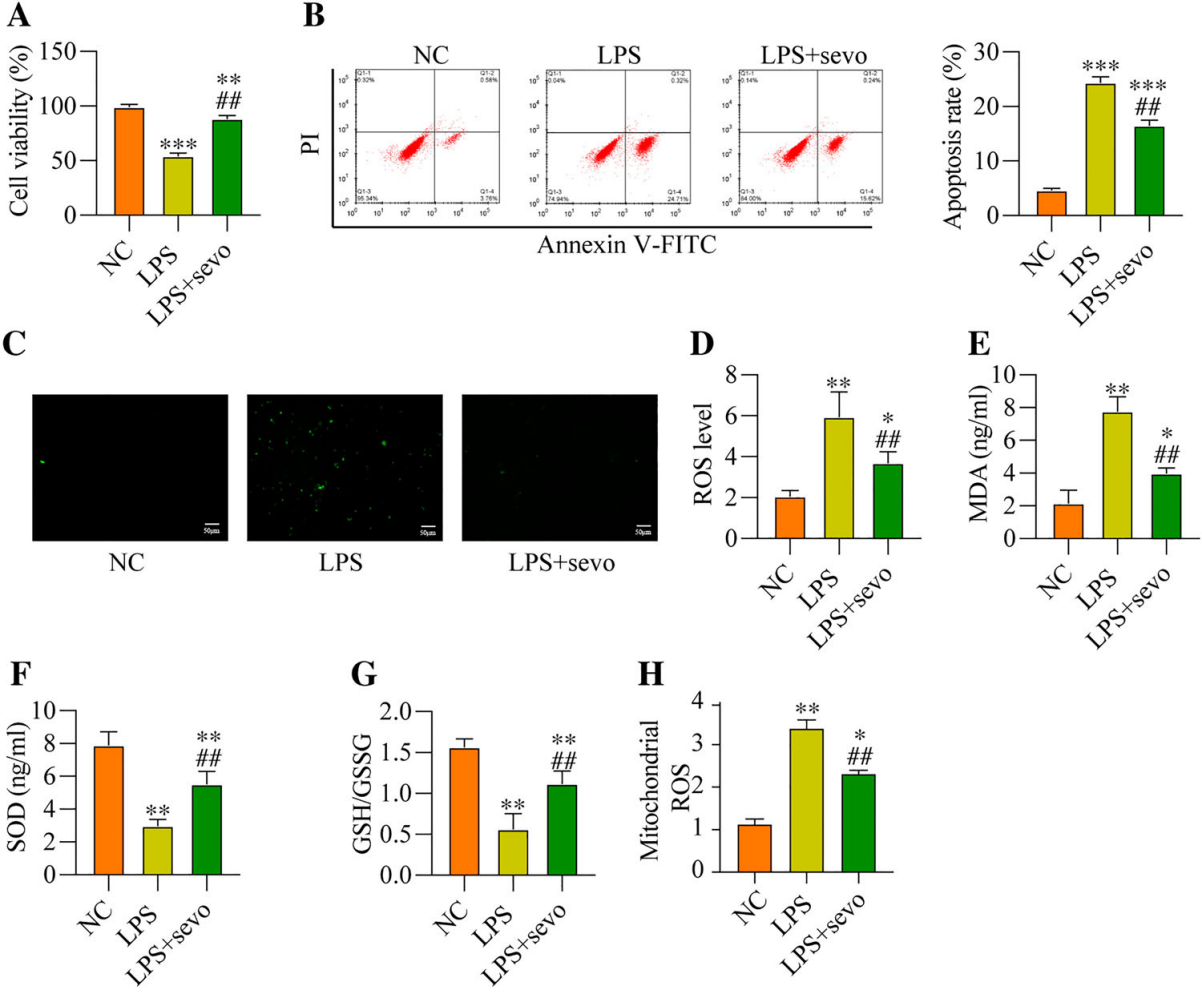
Sevoflurane treatment decreases cytotoxicity and oxidative stress. The RAW264.7 cells were treated with 1 μg/ml LPS stimulation for 2 h, and/or exposed to 3% of sevoflurane for 4 h. Then, (A) Cell viability, (B) Apoptosis rate, (C) DCFH-DA staining, (D) ROS level, (E) MDA level, (F) SOD level, (G) GSH/GSSG ratio, and (H) Mitochondrial ROS level were measured, respectively. All data were presented as the mean ± SD. N = 4 in each group. **P < 0.01 and ***P < 0.001 vs. NC group. ##*P* < 0.01 vs. LPS group. NC, negative control. LPS, Lipopolysaccharide. Sevo, sevoflurane. ROS, reactive oxygen species. MDA, malondialdehyde. SOD, superoxide dismutase. GSH, glutathione. GSSG, oxidized glutathione.

Then, we explored whether sevoflurane also ameliorated LPS-induced cell apoptosis and oxidative stress *in vitro* in a similar manner, thus, the RAW264.7 cells were subjected to LPS treatment and sevoflurane, and were grouped as follows: NC group, LPS group, and LPS plus sevoflurane group. The results in Figure 3(A) suggested that LPS-induced inhibition of cell viability was recovered by co-treating cells with sevoflurane. The following experiments supported that sevoflurane decreased apoptotic cell ratio in the RAW264.7 cells treated with LPS (Figure 3(B)). Then, we discussed the effects of RAW264.7 cells on regulating LPS-induced oxidative damages in the RAW264.7 cells, and the DCFH-DA staining assay expectedly validated that LPS-induced upregulation of cell ROS and mitochondrial ROS level was remarkably abrogated by sevoflurane co-treatment (Figure 3(C–D, H)). Similarly, as shown in Figure 3(E–G), sevoflurane leads to a reduction in the MDA levels, while sevoflurane caused a significant increase in the SOD level and GSH/GSSG ratio. These results suggested that sevoflurane attenuates LPS-induced cytotoxicity and oxidative stress in the RAW264.7 cells *in vitro*.

### The classic anti-oxidant Keap1/Nrf2 pathway can be activated by sevoflurane treatment in the RAW264.7 cells

**Figure 4.**
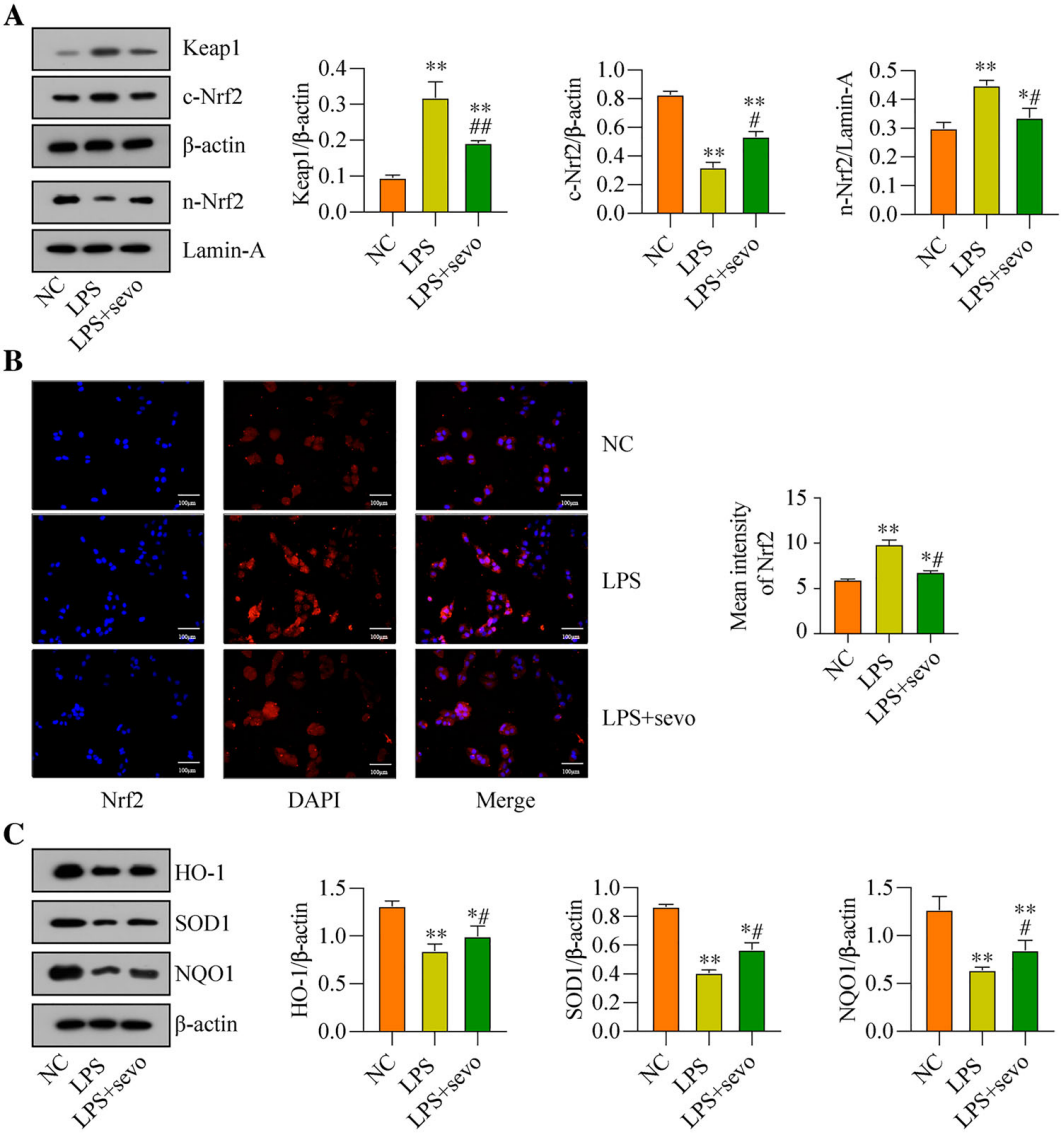
Sevoflurane regulates Keap1-Nrf2 signaling pathway *in vitro*. The RAW264.7 cells were treated with 1 μg/ml LPS stimulation for 2 h and/or exposed to 3% of sevoflurane for 4 h. Then, (A) Keap1 and Nrf2 protein levels, (B) Subcellular localization of Nrf2, and (C) Oxidative stress-associated proteins levels were measured, respectively. All data were presented as the mean ± SD. *N* = 3 in each group. *P < 0.05 and **P < 0.001 vs. NC group. #*P* < 0.05 and ##*P* < 0.01 vs. LPS group. NC, negative control. LPS, Lipopolysaccharide. Sevo, sevoflurane. Keap1, Kelch-like ECH-related protein 1. C-Nrf2, cytoplasmic-nuclear factor erythroid 2-related factor 2, N-Nrf2, nuclear- nuclear factor erythroid 2-related factor 2. HO-1, heme Oxygenase-1. NQO1, NAD(P) H: quinone oxidoreductase 1. SOD1, superoxide dismutase 1.

Next, the potential underlying mechanisms by which sevoflurane ameliorates LPS-induced cytotoxicity in the RAW264.7 cells were explored, and we screened out the classical anti-oxidant Keap1/Nrf2 pathway that was crucial for this process. As previously reported, the Keap1/Nrf2 signaling axis was a critical signaling pathway for regulating oxidative stress [39]. Hence, by performing the Western Blot analysis, we found that LPS promoted upregulation of Keap1, which was suppressed by co-treating cells with sevoflurane (Figure 4(A)). Then, we noticed that sevoflurane increased the expression levels of Nrf2 in the nucleus, whereas the expression levels of cytosolic Nrf2 protein were decreased by sevoflurane in the RAW264.7 cells, as it was respectively validated by the Western Blot analysis (Figure 4(A)) and immunofluorescence staining assay (Figure 4(B)), indicating that sevoflurane promoted Nrf2 translocation from cytoplasm to nucleus. Next, the protein levels of Keap1-Nrf2 pathway-related genes were evaluated, and we found that the protein levels of HO-1, SOD1, and NQO1 were down-regulated with LPS treatment compared with the NC group, whereas sevoflurane led to an up-regulating of HO-1, SOD1 and NQO1 in the LPS + sevoflurane treatment group (Figure 4(C)). These results indicated that sevoflurane activated the anti-oxidant Keap1/Nrf2 pathway in the RAW264.7 cells.

### The Keap1/Nrf2 pathway was involved in sevoflurane-regulated ALI in the mice model

**Figure 5.**
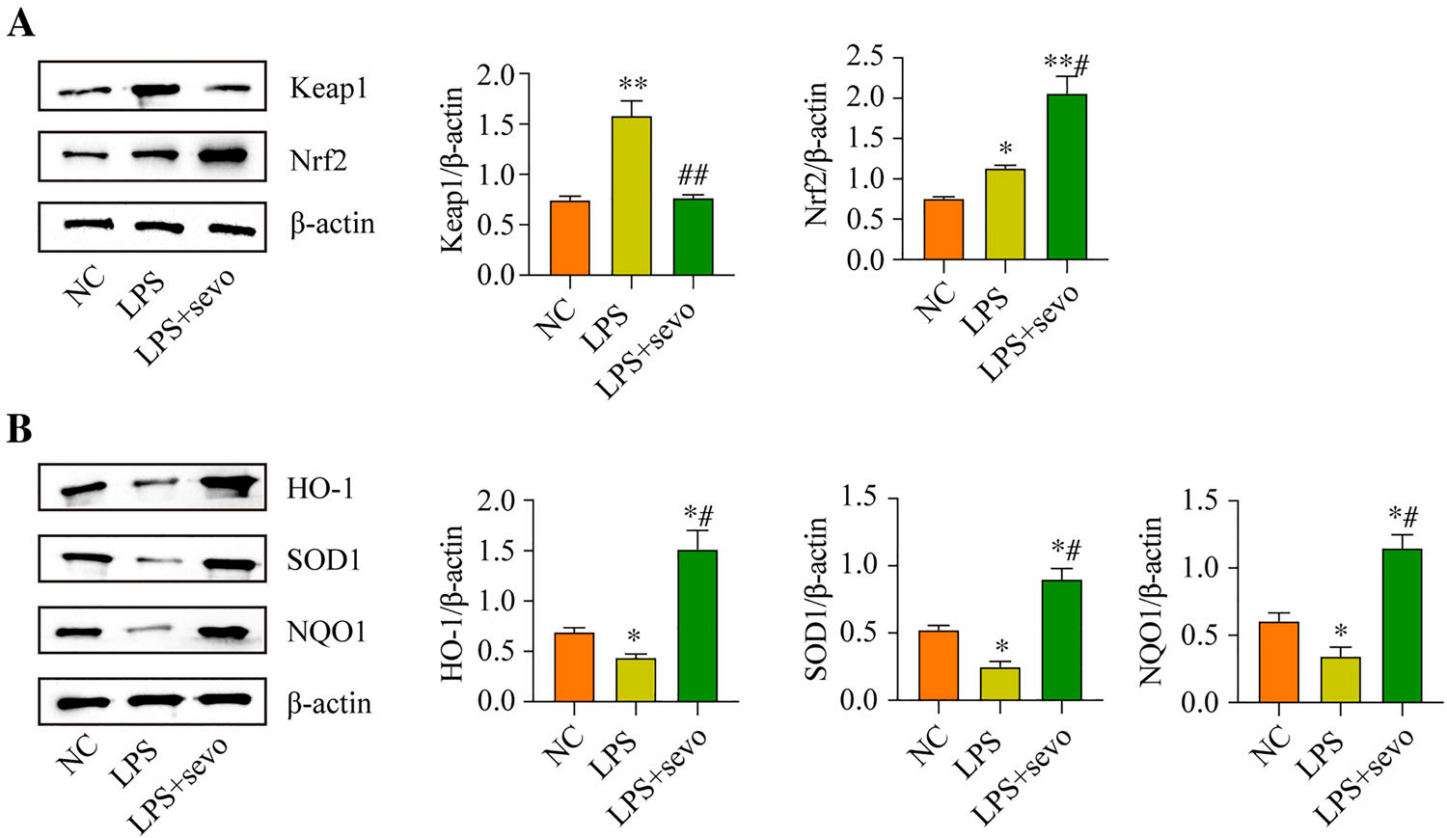
The Keap1/Nrf2 pathway were involved in sevoflurane-regulated ALI in mice model. All mice treated with 5 mg/kg LPS for 2 h and/or administered 3% sevoflurane for 4 h, the lung tissues were collected. Then, (A) Keap1 and Nrf2 protein levels and (B) Oxidative stress-associated proteins levels were measured, respectively. All data were presented as the mean ± SD. *N* = 3 in each group. *P < 0.05 and **P < 0.001 vs. NC group. #*P* < 0.05 and ##*P* < 0.01 vs. LPS group. NC, negative control. LPS, Lipopolysaccharide. Sevo, sevoflurane. Keap1, Kelch-like ECH-related protein 1. C-Nrf2, cytoplasmic-nuclear factor erythroid 2-related factor 2, N-Nrf2, nuclear- nuclear factor erythroid 2-related factor 2. HO-1, heme Oxygenase-1. NQO1, NAD(P) H: quinone oxidoreductase 1. SOD1, superoxide dismutase 1.

To further indicate whether the anti-oxidant Keap1/Nrf2 pathway involves in the sevoflurane-regulated ALI in vivo, we also measured the related protein level in the ALI mice model. We found that LPS increased the Keap1 protein level in the lung tissue, whereas sevoflurane treatment significantly reduced the LPS-induced Keap1 protein level. And the total Nrf2 protein level was increased by sevoflurane treatment (Figure 5(A)). Then, the protein level of Keap1-Nrf2 pathway downstream genes was evaluated. We found that the protein levels of HO-1, SOD1, and NQO1 were decreased with LPS treatment compared with the NC group. But sevoflurane led the protein level of HO-1, SOD1, and NQO1 increased in the LPS + sevoflurane treatment group (Figure 5(B)). The results further showed that sevoflurane was involved in the sevoflurane-regulated ALI.

### Sevoflurane recovers cell viability and restrains oxidative stress in the RAW264.7 cells by activating the Keap1/Nrf2 pathway

**Figure 6.**
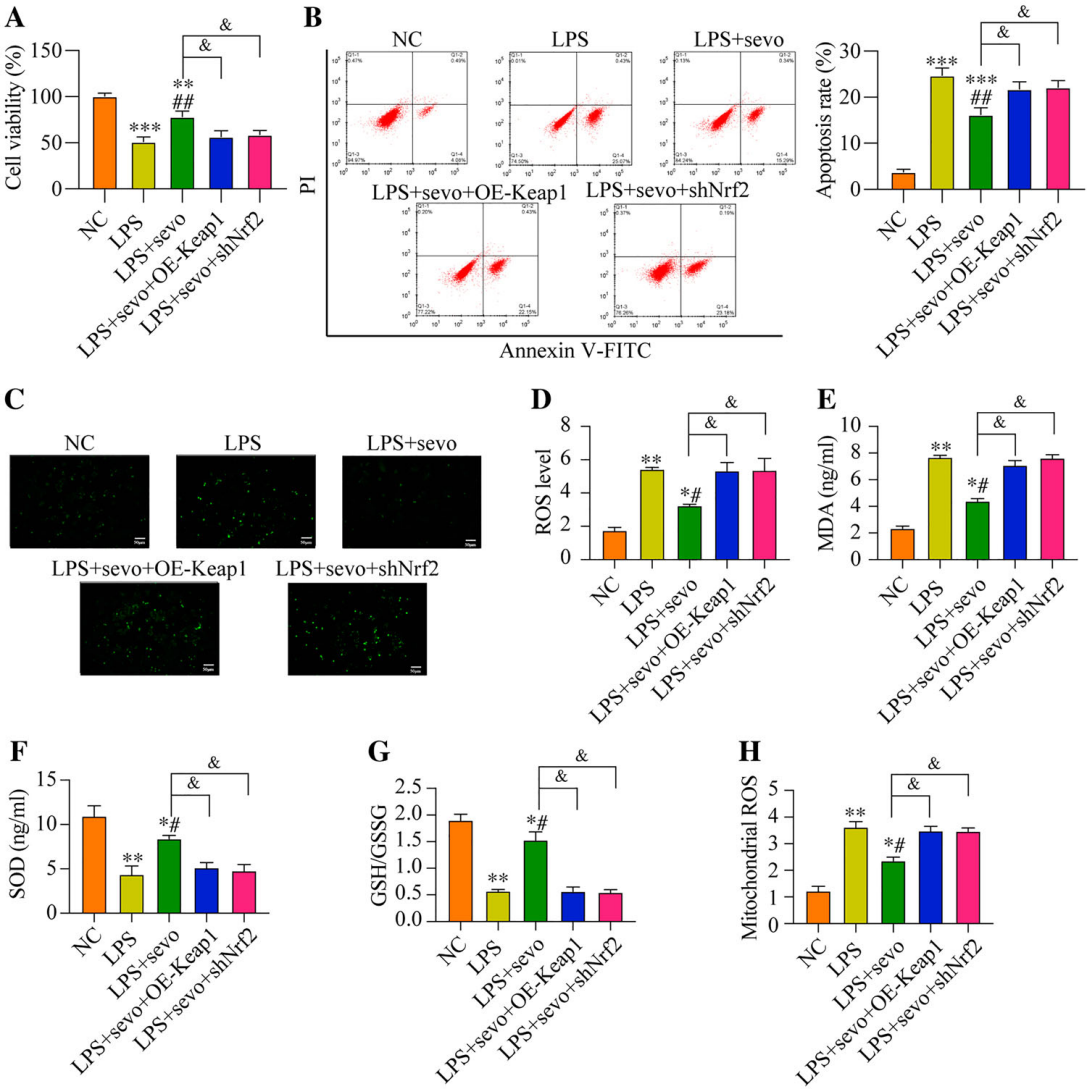
Sevoflurane attenuates cytotoxicity and oxidative stress by regulating Keap1-Nrf2 signaling pathway. The RAW264.7 cells were treated with LPS, 3% of sevoflurane and/or transfected with Keap1 overexpression vector or Nrf2 inhibition vector. Then, (A) Cell viability, (B) apoptosis rate, (C) DCFH-DA staining, (D) ROS level, (E) MDA level, (F) SOD level, (G) GSH/GSSG ratio, and (H) mitochondrial ROS level were measured, respectively. All data were presented as the mean ± SD. *N* = 4 in each group. *P < 0.05 and **P < 0.01 vs. NC group. #*P* < 0.05 and ##*P* < 0.01 vs. LPS group. ^&^*P* < 0.05 vs. LPS + sevo group. NC, negative control. LPS, Lipopolysaccharide. Sevo, sevoflurane. Keap1, Kelch-like ECH-related protein 1. C-Nrf2, cytoplasmic-nuclear factor erythroid 2-related factor 2.

Since our data had evidenced that sevoflurane recovered cell viability in the LPS-treated RAW264.7 cells by eliminating oxidative stress and the critical anti-oxidant Keap1/Nrf2 pathway could also be activated by sevoflurane, which encouraged us to explore whether sevoflurane suppressed LPS-induced cell death through the Keap1/Nrf2 pathway. To validate this hypothesis, Keap1 was overexpressed, whereas Nrf2 was silenced in the RAW264.7 cells, and the transfection efficiencies of Keap1 and Nrf2 were presented in Figure S3A-B. Specifically, compared with NC and empty vector group, the Keap1 protein level was up-regulating in the pcDNA-Keap1 group (Figure S3A), while the Nrf2 protein level was down-regulating in the shRNA-Nrf2 group (Figure S3B). As it was shown in Figure 6(A), sevoflurane recovered cell viability in the LPS-treated RAW264.7 cells, which were abrogated by both Keap1 overexpression and Nrf2 ablation. In addition, the Annexin V-FITC/PI double staining assay results verified that both Keap1 overexpression and Nrf2 downregulation significantly increased cell apoptosis ratio in the RAW264.7 cells co-treated with sevoflurane and LPS (Figure 6(B)). Then, we also verified the effect of the Keap1-Nrf2 pathway on regulating LPS-induced oxidative damages in RAW264.7 cell. The results suggested that the ROS level and MDA level were decreased in the sevoflurane + LPS group compared with the LPS group. But comparing with the sevoflurane + LPS group, the ROS level, MDA level, and the mitochondrial ROS level were increased by Keap1 overexpression or Nrf2 ablation (Figure 6(C–E, H)). Moreover, Keap1 overexpression or Nrf2 downregulation remarkable reduced the SOD level and GSH/GSSG ratio in the RAW2264.7 cells co-treated with sevoflurane and LPS (Figure 6(F–G)). The above results suggested that sevoflurane ameliorated LPS-induced apoptotic cell death and oxidative stress in the RAW264.7 cells through activating the Keap1/Nrf2 pathway.

### Local Keap1 overexpression or Nrf2 inhibited aggravated ALI in mouse

**Figure 7.**
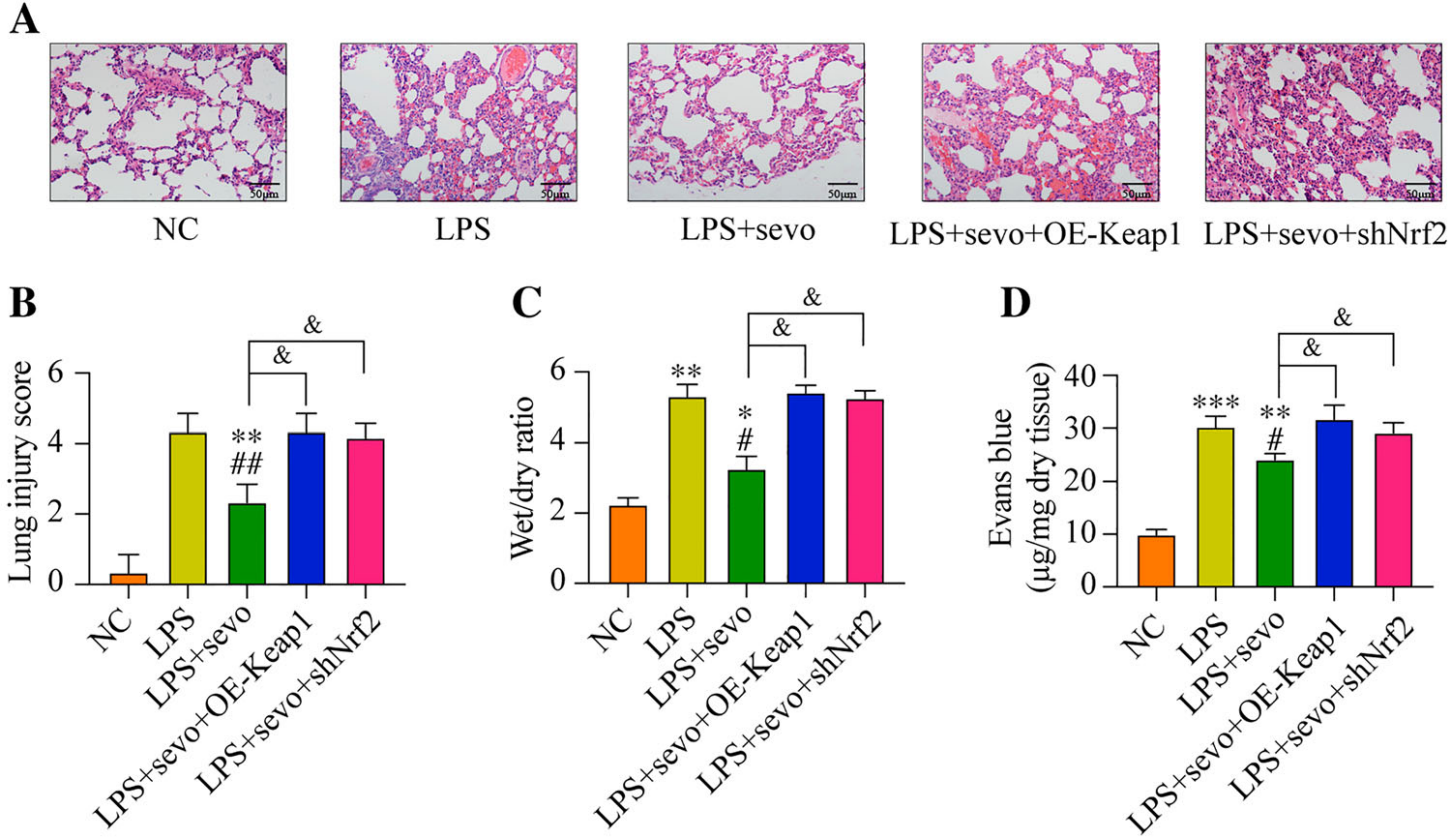
Sevoflurane attenuates ALI by regulating Keap1-Nrf2 signaling pathway. All mice treated with LPS, 3% sevoflurane, and/or local transfected with Keap1 overexpression vector or Nrf2 inhibition vector. Then the lung tissues were collected. (A) The histology of lung tissues, (B) lung injury score, (C) wet/dry of lung tissues, and (D) permeability of lung vasculature were measured, respectively. All data were presented as the mean ± SD. *N* = 6 in each group. *P < 0.05 and **P < 0.01 vs. NC group. #*P* < 0.05 and ##*P* < 0.01 vs. LPS group. ^&^*P* < 0.05 vs. LPS + sevo group. NC, negative control. LPS, Lipopolysaccharide. Sevo, sevoflurane. Keap1, Kelch-like ECH-related protein 1. C-Nrf2, cytoplasmic-nuclear factor erythroid 2-related factor 2.

Finally, we examined whether local Keap1 overexpression or Nrf2 inhibition was able to affect the ALI in vivo. Sevoflurane attenuated the inflammatory cells infiltration and thickened the alveolar walls in mice lung tissues. However, the lung inflammation was aggravated by both Keap1 overexpression or Nrf2 inhibited (Figure 7(A–B)). Moreover, compared with the LPS plus sevoflurane treatment, both Keap1 overexpression and Nrf2 downregulation significantly increased the lung wet/dry ratio and Evans blue permeability. These data indicated that exogenous regulation of Keap1/Nrf2 is a significant mechanism to control sevoflurane-alleviated ALI.

## Discussion

To date, the volatile anesthetic sevoflurane has shown protective effects in the treatment of ALI, and previous publications indicate that sevoflurane alleviates ALI by inhibiting excessive inflammation and apoptosis [40,41]. In addition, several researches have showed that oxidative stress involves in promoting ALI progression. For example, Imai et al. [42] demonstrate that oxidative damages contribute to ALI, and other researchers find that inhibition of ROS significantly recover pulmonary functions in ALI [43], suggesting that targeting oxidative stress may be novel strategy to treat ALI. Thus, it is urgent to search for anti-oxidant drugs which may be the ideal therapeutic agent for ALI. Interestingly, previous data support that sevoflurane exerts its anti-oxidant effects to remove ROS stresses, and protects cells from oxidative damages-induced cell death. However, it is still unclear whether sevoflurane-eliminated oxidative stress to ameliorate ALI. Based on the above-existed information, this study established ALI models by using the LPS treatment method, and we verified that both oxidative stress and apoptosis contributed to the aggravation of LPS-induced ALI. Of note, we found that sevoflurane ameliorates LPS-induced ALI, and restrained LPS-induced ROS generation and apoptosis, suggesting that sevoflurane attenuated LPS-induced ALI was associated with oxidative stress and apoptosis.

Moderate oxidative stress-triggered ROS generation is a normal biological process to sustain the balance in human body and cells, however, recent data illustrate that excessive generation of ROS and oxygen radicals contribute to pathological changes and disease development, and recent data show that aberrant ROS generation promotes super-inflammation and endothelial permeability to accelerate tissue damages [43], supporting the fact that oxidative stress plays dual-role in regulating biological functions. Of note, it has been reported that sevoflurane reduces oxidative stress in some diseases. For example, Wu et al. [44] show that sevoflurane ameliorates ischemia/reperfusion injury by eliminating ROS, and other study support that sevoflurane also suppress oxidative stress to rescue neural functions under hypoxic stresses [45]. In this study, we similarly found that sevoflurane treatment could decrease the oxidative stress in LPS-induced in vitro and in vivo ALI models, including the reduction of cell ROS and MDA levels and the increase in the SOD level. GSH is deemed as a critical ROS scavenger, which is transformed into GSSG to eliminate ROS, thus, the GSH/GSSG ratio is often used as a biomarker to evaluate the extent of oxidative stress [38]. Interestingly, our study showed that sevoflurane treatment recovered GSH/GSSG ratio in LPS-treated ALI models. Mitochondria have been suggested to be the primary origin of oxidative stress via the overproduction of ROS. Mitochondria are responsible for producing ATP through oxidative phosphorylation (OXPHOS) and have a calcium buffering capacity for the cell. Defects in mitochondria will lead to declined antioxidant capacity and cell apoptosis [46]. In this study, the mitochondrial ROS level was reduced by sevoflurane treatment. Accordingly, the cell apoptosis ratio also decreased. These findings demonstrated that sevoflurane decreased ROS levels to attenuate LPS-induced ALI, however, further researches are needed to completely understand the possible molecular mechanisms.

As previously reported, the classical Keap1/Nrf2 pathway is pivotal for regulating oxidative stress, and in oxidative stress conditions, the activated Nrf2 translocates from cytoplasm to nucleus to activate the downstream anti-oxidant effectors through binding to ARE [47]. Also, Nrf2 is reported to act as an organizer for multiple signaling pathways, which is reported to regulate both inflammation and oxidative damages. Based on the above information, we evidenced that sevoflurane treatment could effectively up-regulated nuclear-Nrf2 protein expression induced by LPS in total cell lysates, whereas the Keap1 and Nrf2 protein levels in the cytoplasm were down-regulated, suggesting that the anti-oxidant Keap1/Nrf2 pathway could be activated by sevoflurane. Also, we discussed whether sevoflurane-alleviated LPS-induced cytotoxicity and ALI by regulating the Keap1/Nrf2 pathway *in vitro* and *in vivo*, and the results expectedly showed that sevoflurane rescued cell viability in the LPS-treated RAW264.7 cells by activating the Keap1/Nrf2 pathway to eliminate ROS generation. Furthermore, exogenous regulation of Keap1 and Nrf2 also affects the lung injury degree. These all results suggested that sevoflurane reduces oxidative stress and regulates the Keap1-Nrf2 signaling pathway to attenuate LPS-induced ALI. Although we had preliminarily investigated the role and potential underlying mechanisms by which sevoflurane exerted its protective effects on LPS-induced ALI, the deep mechanisms by which sevoflurane inhibited LPS-induced oxidative stress through regulating other signaling pathways in addition to the Keap1/Nrf2 pathway was not fully understood, which needed to be further studied in our future research.

## Conclusions

In conclusion, our findings firstly indicated that sevoflurane effectively protected ALI against oxidative stress, which was dependent on the regulation of the Keap1/Nrf2 pathway. Therefore, combined with previous studies, sevoflurane possesses potent antioxidant properties in ALI. This study evidenced that sevoflurane could potentially be used as an anti-oxidant and anti-inflammatory drug for the treatment of ALI in clinical practices.

## Supplementary Material

Supplemental Material

Supplemental Material

Supplemental Material

## Data Availability

The authors confirm that the data supporting the findings of this study are available within the article and its supplementary materials.
