# Supplementary figures and images for: Sevoflurane reduces lipopolysaccharide-induced apoptosis and pulmonary fibrosis in the RAW264.7 cells and mice models to ameliorate acute lung injury by eliminating oxidative damages
Source: Redox Rep. 2022 Jul 8;27(1):139–49. doi: 10.1080/13510002.2022.2096339 (PMC9272930; doi:10.1080/13510002.2022.2096339)

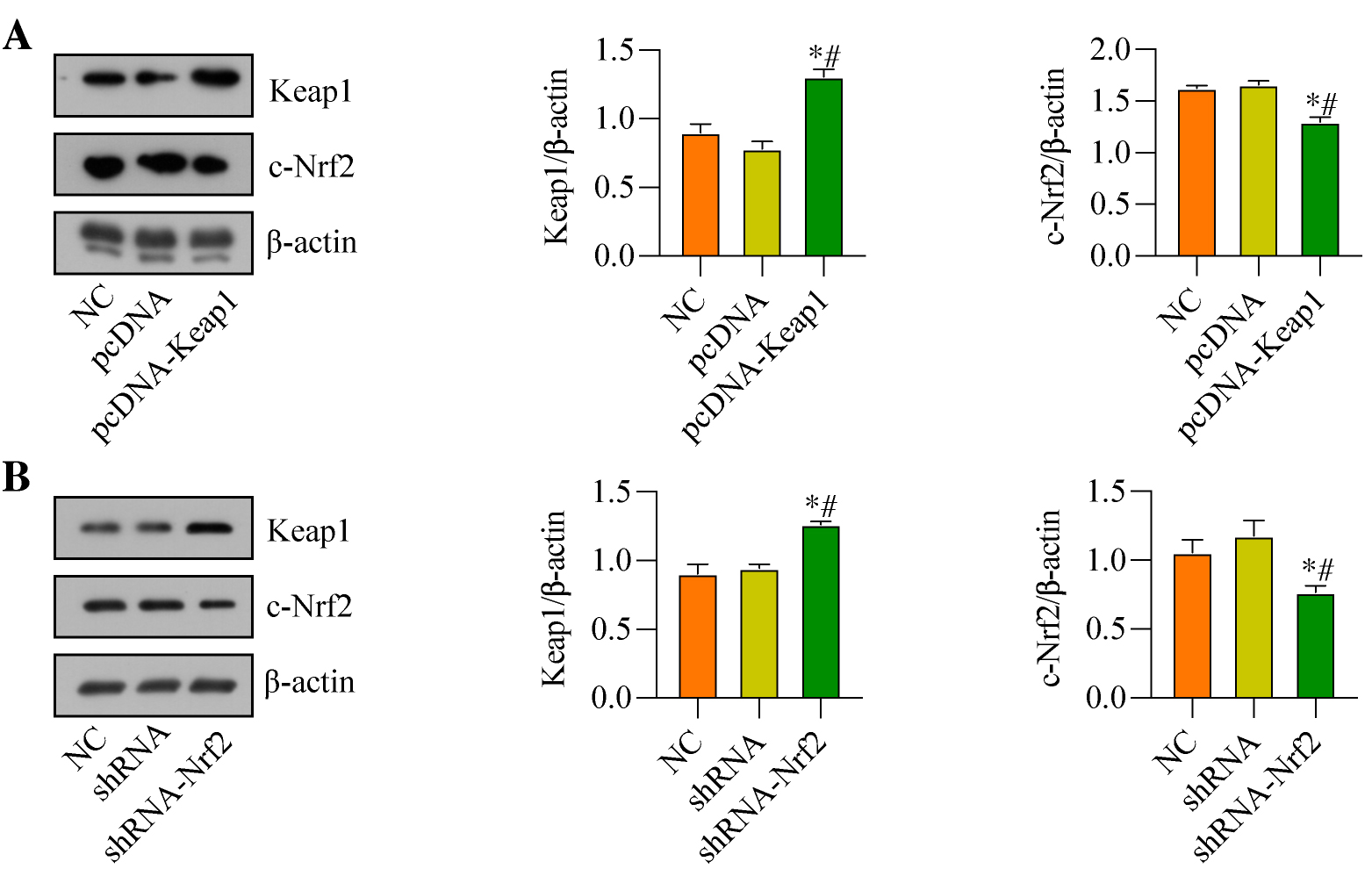

Supplement: Supplemental Material [file YRER_A_2096339_SM5224.jpg]

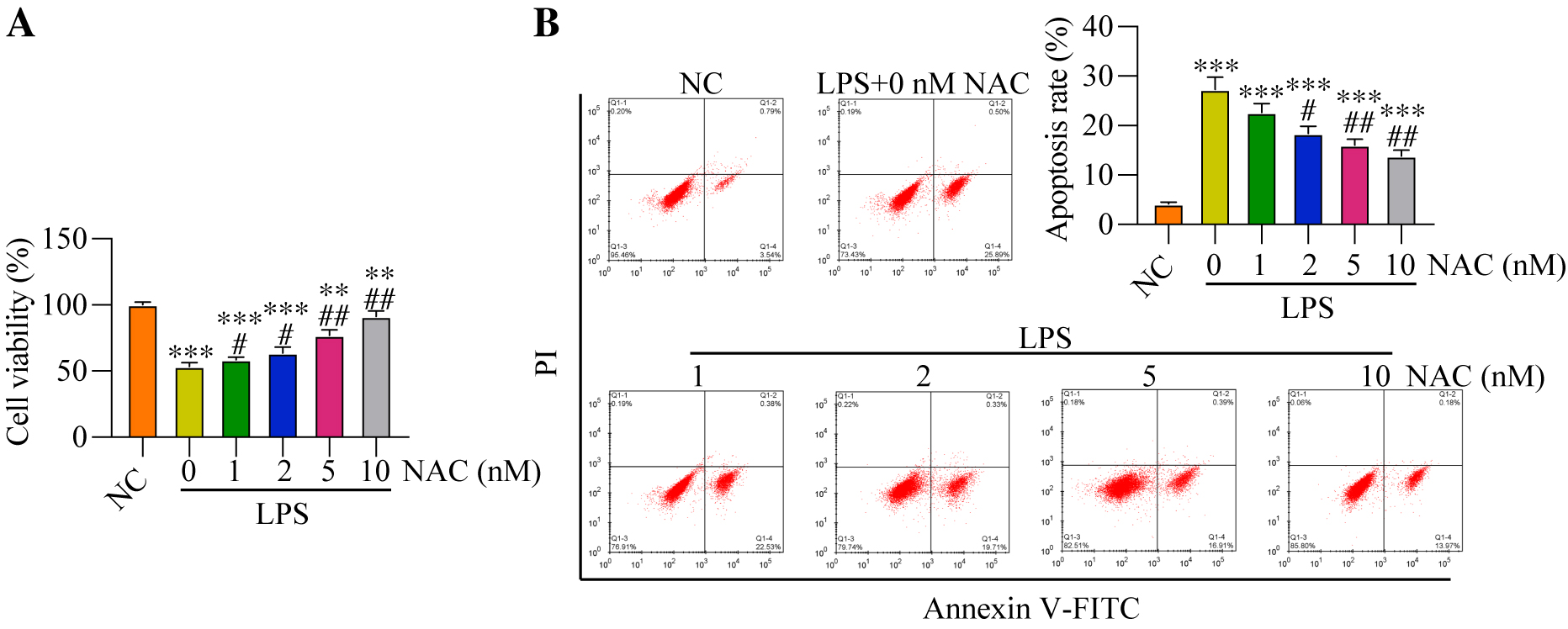

Supplement: Supplemental Material [file YRER_A_2096339_SM5221.jpg]

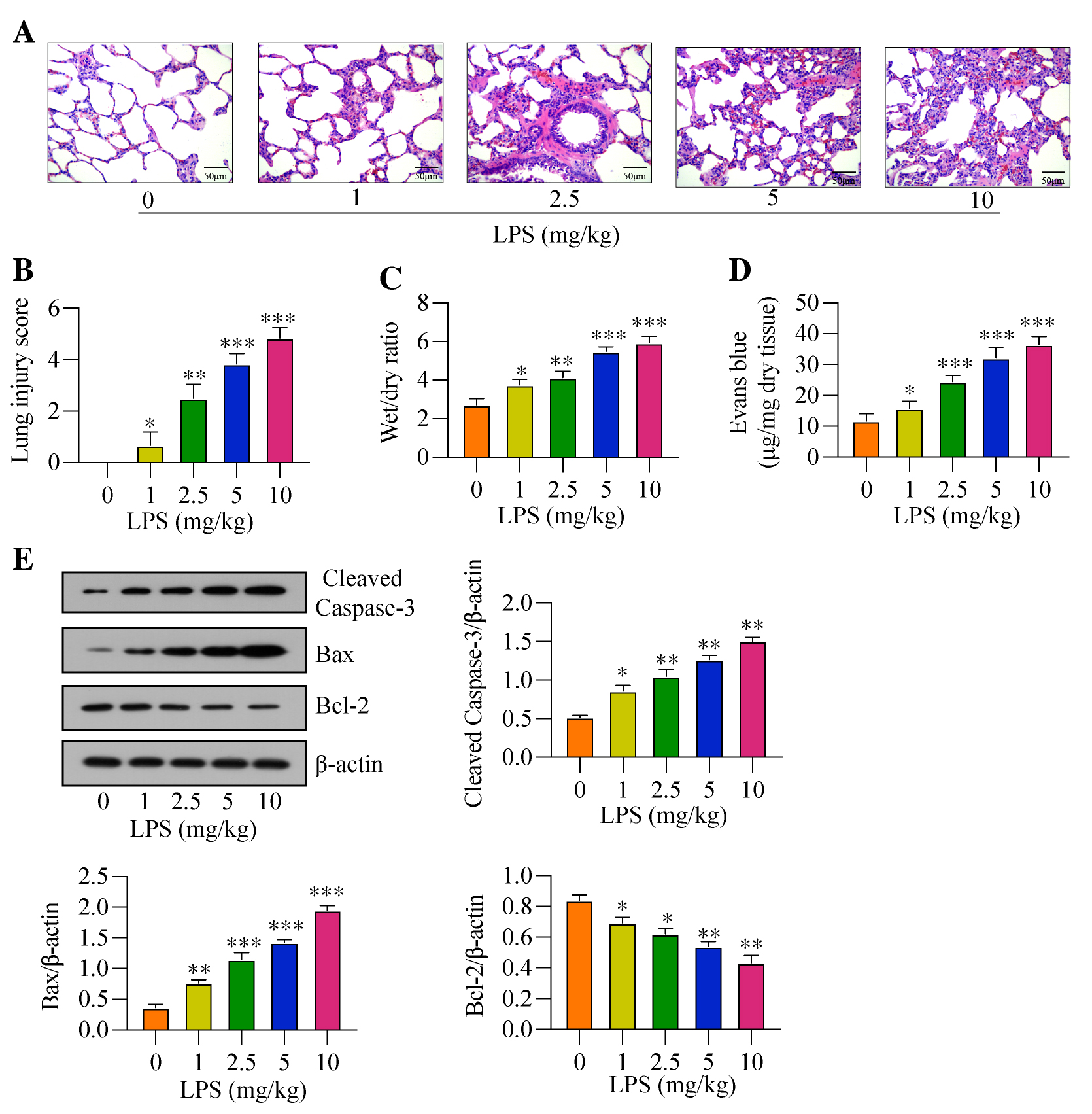

Supplement: Supplemental Material [file YRER_A_2096339_SM5218.jpg]
